# Multicomponent Modification of Polyethylene-Based Films for Enhanced Hydrophobicity and Balanced Gas Transport and Water Vapor Barrier Properties

**DOI:** 10.3390/membranes16100316

**Published:** 2026-09-25

**Authors:** Lijing Duan, Shuaiqi Fang, Leying Sun, Yawen Wang, Haobo Xiu, Huabin Wang, Abin Chen, Baoxiu Wang, Shiqiang Song, Zhenlin Jiang

**Affiliations:** 1 College of Chemistry and Chemical Engineering, Research Center for Advanced Mirco- and Nano-Fabrication Materials, Shanghai University of Engineering Science, Shanghai 201620, China; dlj18211887619@163.com (L.D.);; 2 Baihong Polyester Fiber Technology Industrial Co., Ltd., Fenglin Industrial Zone, Longhu Town, Jinjiang 362241, China; 3 College of Materials Science and Engineering, Donghua University, Shanghai 201620, China; 4 Hunan Meilape Technology Development Co., Ltd., Changsha 410100, China

**Keywords:** polyethylene composite membrane, hydrophobic film, CO_2_ transport, water vapor barrier, fluorinated polymer, silica nanoparticles

## Abstract

Hydrophobic gas-permeable polyethylene films with balanced water vapor resistance and gas transport properties are desirable for membrane applications; however, achieving an effective balance between moisture barrier performance and gas permeability remains challenging. In this work, dense non-porous polyethylene-based composite films were fabricated through melt blending and film casting by incorporating fluorinated ethylene propylene copolymer (FEP), polyethylene wax (PE wax), and hydrophobic silica nanoparticles (SiO_2_). The effects of multicomponent modification on surface properties, crystallization behavior, morphology, and transport performance were investigated. The optimized formulation, PEH-2, containing 3 wt% PE wax and 1 wt% hydrophobic SiO_2_ based on the LLDPE/POE matrix, exhibited the most balanced transport performance. The synergistic regulation of surface energy, interfacial morphology, and diffusion pathways by FEP, PE wax, and SiO_2_ enhanced hydrophobicity while maintaining CO_2_ transport capability. The dense composite structure increased resistance to water vapor diffusion, whereas regulated molecular pathways facilitated CO_2_ transport. PEH-2 exhibited a water contact angle of 110.75°, a CO_2_ transmission rate of 6498 cm^3^·m^−2^·day^−1^ at 0.1 MPa, and a WVTR of 1.20 g·m^−2^·day^−1^. The CO_2_ transmission rate/WVTR ratio (approximately 5.4 × 10^3^) was used to evaluate the balance between gas transport and moisture barrier performance. This study provides a scalable strategy for designing polyethylene-based composite membranes with balanced moisture resistance and gas transport properties.

## 1. Introduction

Polyethylene (PE)-based hydrophobic functional films with selective gas transport and water resistance have attracted considerable attention due to their potential in membrane applications [1,2,3,4]. Owing to their excellent chemical stability, low cost, and scalable processability, these membranes have been widely applied in protective materials, packaging, and moisture management systems. In practical applications, hydrophobic breathable membranes are expected to provide efficient moisture dissipation while maintaining effective moisture protection. However, conventional polyethylene membranes still suffer from an intrinsic trade-off between water resistance and gas permeability. Enhancing water resistance often requires dense structures or low-surface-energy modifications, which inevitably restrict gas transport, whereas increasing permeability through porous structures tends to compromise liquid water resistance and mechanical durability. Therefore, developing PE-based films that simultaneously achieve high water vapor resistance and efficient gas transport remains a critical challenge for advanced functional polymer materials.

To overcome the limitations of conventional PE membranes, various strategies have been explored to regulate surface chemistry and internal membrane architectures [1,5,6,7]. Generally, the design of hydrophobic breathable membranes can be categorized into three major approaches: surface energy regulation, porous structure engineering, and multifunctional composite modification. Surface modification strategies mainly focus on introducing low-surface-energy components, such as fluorinated polymers, silane derivatives, or silicone-based materials, to reduce surface wettability and improve water repellency [8,9,10]. Although these approaches can effectively enhance hydrophobicity, excessive surface modification may block molecular transport pathways and reduce gas permeability, while complicated chemical treatments often limit large-scale manufacturing. Alternatively, constructing hierarchical porous structures has been widely investigated to improve gas permeability by providing continuous transport channels. Techniques including phase separation, stretching-induced pore formation, electrospinning, and template-assisted methods enable the fabrication of membranes with controllable pore size and porosity. However, precisely balancing pore dimensions is challenging because enlarged pores may facilitate vapor transmission but simultaneously increase the risk of liquid water penetration. Recently, melt blending has emerged as an attractive strategy for preparing functional polymer membranes because of its simple processing, environmental advantages, and compatibility with industrial polymer manufacturing [2,11,12]. By incorporating inorganic nanoparticles, fluorinated polymers, or crystallization regulators into polymer matrices, the surface morphology, phase structure, and transport behavior of membranes can be simultaneously tailored.

Among various functional additives, silica nanoparticles have attracted considerable attention in polymer membrane systems due to their ability to regulate interfacial morphology and molecular diffusion pathways. The incorporation of silica nanoparticles can generate polymer–filler interfaces and increase the tortuosity of transport pathways, thereby influencing gas and vapor transport behavior [13,14,15]. However, the aggregation tendency of inorganic nanoparticles within nonpolar polyethylene matrices remains a challenge, which may limit their effectiveness. Fluorinated polymers, such as fluorinated ethylene propylene copolymer (FEP), are widely used for hydrophobic modification because of their low surface energy fluorocarbon groups. The introduction of fluorinated segments can reduce surface wettability and improve water repellency of polymer films. Nevertheless, excessive incorporation of fluorinated components may cause phase separation or poor interfacial compatibility with polyethylene. Polyethylene wax, as a low-molecular-weight polyolefin additive, can influence polymer processing behavior, chain mobility, and crystallization characteristics. In polyethylene-based composite systems, PE wax can improve melt processability and facilitate the dispersion of functional additives.

However, single-component modification strategies usually improve only specific aspects of membrane performance, making it difficult to simultaneously achieve desirable water vapor resistance, gas permeability, and mechanical stability. Therefore, increasing attention has been paid to multicomponent modification approaches, in which different functional components are introduced to regulate surface properties, interfacial morphology, and transport behavior of polymer membranes. Such integrated strategies provide opportunities to improve the balance between water resistance and gas transport performance. Nevertheless, achieving an optimized multicomponent structure through a simple and scalable fabrication process remains challenging, particularly for polyethylene-based systems due to the nonpolar nature of the matrix and limited compatibility with functional additives. Consequently, developing an effective multicomponent modification strategy for polyethylene membranes is highly desirable.

In this work, a multicomponent modification strategy was developed to fabricate polyethylene-based functional films through melt blending and film casting. Polyethylene wax (PE wax), fluorinated ethylene propylene copolymer (FEP), and hydrophobic silica nanoparticles (SiO_2_) were simultaneously incorporated into an LLDPE/POE matrix to regulate surface properties, interfacial morphology, and transport behavior. The synergistic effects of FEP, PE wax, and SiO_2_ on hydrophobicity, crystallization behavior, interfacial structure, and gas transport characteristics were systematically investigated. This work aims to establish the relationship between multicomponent modification, structural evolution, and transport properties, providing a scalable strategy for designing polyethylene-based composite membranes with balanced water vapor resistance and gas transport performance.

## 2. Materials and Methods

### 2.1. Materials

Linear low-density polyethylene (LLDPE, grade 41E687) was supplied by Dow Chemical Company (Midland, MI, USA). Polyolefin elastomer (POE, grade DF740) and polyethylene wax (PE wax, grade 07500) were purchased from Mitsui Chemicals, Inc. (Tokyo, Japan). Fluorinated ethylene propylene copolymer (FEP, grade CN120) was provided by DuPont (Wilmington, DE, USA). Hydrophobic silica nanoparticles (SiO_2_, HDK^®^ H20, Wacker Chemie AG, Munich, Germany) were used as inorganic fillers. HDK^®^ H20 is a hydrophobic fumed silica with surface-treated silanol groups, which facilitates its compatibility with the nonpolar polyethylene matrix. The average primary particle size of HDK^®^ H20 is approximately 20 nm. Diiodomethane (analytical grade, 99%) and absolute ethanol (analytical grade, ≥99.99%) were purchased from Macklin Biochemical Technology Co., Ltd. (Shanghai, China). All materials were used as received without further purification.

### 2.2. Preparation of PE Wax/FEP/SiO_2_ Synergistic Polyethylene Films

The fabrication process of polyethylene-based functional films is schematically illustrated in Figure 1. Briefly, linear low-density polyethylene (LLDPE), polyolefin elastomer (POE), polyethylene wax (PE wax), hydrophobic silica nanoparticles (SiO_2_), and fluorinated ethylene propylene copolymer (FEP) were premixed in a high-speed mixer for 3–5 min according to the designed formulations (Table 1). The contents of PE wax and SiO_2_ were selected as 1 wt% and 3 wt% based on considerations of composite processability and interfacial regulation. Low concentrations of silica nanoparticles are generally preferred in polymer nanocomposites because they can regulate interfacial structures and may influence molecular diffusion pathways while minimizing particle aggregation. Meanwhile, moderate PE wax incorporation can improve melt processability and promote the dispersion of functional additives without significantly affecting the integrity of the polyethylene matrix. Therefore, two representative loading levels (1 wt% and 3 wt%) were selected to investigate the effects of additive content on the morphology, crystallization behavior, surface wettability, and transport properties of the composite films [16,17,18,19]. The premixed materials were subsequently melt blended using a co-rotating twin-screw extruder at temperatures of 265, 280, 275, and 270 °C from the feeding zone to the die zone, respectively. The extruded strands were rapidly cooled in a water bath, air-dried, and pelletized to obtain the composite granules.

The obtained pellets were dried at 80 °C for 1 h and subsequently processed into films through a film casting extrusion process. During film formation, the molten composites were extruded through a flat die and cooled on a casting roller to obtain the final polyethylene-based composite films. The prepared samples were denoted as PEF and PEH-x, where the detailed compositions of LLDPE, POE, FEP, PE wax, and SiO_2_ are summarized in Table 1. Further details of the experimental procedure are provided in Appendix A.

### 2.3. Characterization

#### 2.3.1. Structural and Morphological Characterization

The chemical structures of the films were characterized by Fourier-transform infrared spectroscopy (FTIR, Nicolet 8700, Thermo Fisher Scientific, Wilmington, NC, USA) over the range of 500–4000 cm^−1^ at a resolution of 4 cm^−1^. The three-dimensional surface morphology and height variations in the cast films were analyzed using a laser scanning confocal microscope (CLSM, VK-X3000, KEYENCE, Tokyo, Japan) equipped with a 10× objective lens (numerical aperture of 0.25). The surface height profiles were obtained from CLSM images, and the arithmetic mean height deviation (Sa) was used to quantitatively describe the surface topographical variation. The *Z*-axis scanning step and laser power were set to 0.1 μm and 40%, respectively. The surface and cross-sectional morphologies of the films were further observed using field-emission scanning electron microscopy (FE-SEM, Hitachi SU8010, Tokyo, Japan) at an accelerating voltage of 15 kV after gold sputter coating. The elemental distribution was analyzed using an Oxford Instruments X-Max 50 energy-dispersive X-ray spectroscopy (EDS) detector (Oxford Instruments, High Wycombe, UK). The elemental mapping analysis was performed on the film surface to evaluate the spatial distribution of fluorine- and silicon-containing regions.

#### 2.3.2. Thermal and Crystallization Behavior

The crystallization and thermal behaviors of the composite films were investigated using differential scanning calorimetry (DSC, JKSB009, Waltham, MA, USA) under a nitrogen atmosphere (50 mL min^−1^). Film samples (~5 mg) were heated from 20 to 300 °C at a heating rate of 10 °C min^−1^, held for 10 min to eliminate thermal history, cooled to −30 °C, and subsequently reheated to 300 °C at the same rate. The crystallinity ($X_{c}$) of the LLDPE phase was calculated according to Equation (1).

(1) $$X_{c}=\frac{\Delta H_{m}}{\Delta {H_{m}}^{0}\times W_{LLDPE}}\times 100\%$$

where $\Delta H_{m}$ is the measured heat of fusion of the LLDPE phase, $\Delta {H_{m}}^{0}$ is the heat of fusion of 100% crystalline polyethylene (293 J g^−1^), and W_LLDPE_ is the mass fraction of LLDPE in the composite formulation (0.70).

#### 2.3.3. Rheological Properties

The melt-processing behaviors of the composite materials were evaluated using a melt flow index tester (HS-XNR-400A, HESON, Shanghai Hesheng Instrument Technology Co., Ltd., Shanghai, China) and a torque rheometer (CTR-T200, CHANGKAI, Shanghai Changkai M&E Technology Co., Ltd., Shanghai, China). The melt flow rate (MFR) was measured at 190 °C under a load of 2.16 kg after a 300 s preheating period. Torque measurements were conducted at 270 °C and 80 rpm after 5 min of preheating.

#### 2.3.4. Film Thickness and Optical Properties

The thickness of the cast films was measured using a digital micrometer at randomly selected positions, and the average thickness value was used for permeability calculations.

Optical transmittance was measured using a UV-Vis spectrophotometer (Lambda 1050, PerkinElmer, Waltham, MA, USA) over the wavelength range of 50–800 nm with a resolution of 1 nm. The haze values of the films were determined using a haze meter (YH1200) according to GB/T 2410-2008 [20]. The average thickness of the prepared films was approximately 150 μm.

#### 2.3.5. Surface Wettability Characterization

The static water contact angle of the film surfaces was measured using a contact angle analyzer (DSA30S) with 2 μL droplets of ultrapure water and diiodomethane. The surface free energy of the films was calculated using the Owens–Wendt–Rabel–Kaelble (OWRK) method.

#### 2.3.6. Mechanical Properties

The mechanical properties of the films were evaluated using a universal testing machine (AGS-X, Shimadzu, Kyoto, Japan) according to GB/T 1040.3-2022. Dumbbell-shaped film specimens (150 mm × 20 mm × 0.15 mm) were tested at a crosshead speed of 50 mm min^−1^ after conditioning for 24 h.

#### 2.3.7. Gas Transport Properties

The water vapor transmission rate (WVTR) was measured using a W3/030 water vapor transmission rate tester (Labthink, Jinan, China). Before testing, the film samples were conditioned at 23 ± 0.5 °C and 50 ± 2% relative humidity (RH) for 48 h. Measurements were performed at 38 °C under a humidity gradient of 90% RH/50% RH with a sealing pressure of 0.8 MPa and an effective permeation area of 16.27 cm^2^. Four independent measurements were performed for each sample, and the average value was used for analysis.

The CO_2_ permeability of the films was measured using a differential-pressure gas permeability tester (GDP-C, Labthink, China) according to GB/T 1038-2000 [21]. Before testing, samples were conditioned at 23 ± 0.5 °C and 50 ± 2% RH for 48 h. The measurements were performed at 23 °C under dry conditions with a pressure difference of 0.1 MPa.

All measurements were performed using at least three independent specimens, and the results are reported as mean values with standard deviations.

## 3. Results and Discussion

### 3.1. Structural Characterization and Interfacial Morphology of PE Composite Films

Figure 2 presents the chemical structure, surface topographical features, and elemental distribution of the polyethylene-based composite films. As shown in Figure 2A, the FTIR spectra demonstrate the incorporation of FEP and hydrophobic SiO_2_ into the PE matrix. After introducing SiO_2_, the absorption bands located at approximately 1095 and 798 cm^−1^ become more pronounced, which are assigned to the asymmetric and symmetric stretching vibrations of Si–O–Si bonds, respectively. These characteristic signals indicate the presence of silica nanoparticles in the composite films. In addition, the characteristic absorption bands associated with the polyethylene matrix are observed, including the C–H stretching vibrations in the range of 2800–3000 cm^−1^ and C–H deformation vibrations near 1460 cm^−1^. The FTIR results confirm that the functional components were successfully incorporated into the PE matrix during melt blending.

The absorption band near 1360 cm^−1^ is associated with the C–H deformation vibrations of hydrocarbon components, including polyethylene and PE wax. In addition, the absorption band at approximately 1151 cm^−1^ is assigned to the stretching vibration of C–F bonds in FEP, while the weak feature near 980 cm^−1^ may be related to fluorinated groups. These characteristic absorption bands indicate the presence of FEP-related structures in the composite films [19,22,23]. No new absorption peaks associated with chemical bond formation are observed after the incorporation of FEP, PE wax, and SiO_2_, suggesting that the components were physically blended into the PE matrix without detectable chemical reactions during melt processing. Due to the similar molecular structure of PE wax and polyethylene, their characteristic absorption bands overlap significantly, making the independent identification of PE wax from the FTIR spectra difficult.

The three-dimensional surface morphology and topographical variation in the composite films were further evaluated by confocal laser scanning microscopy (CLSM), as shown in Figure 2B. The arithmetic mean height deviation (Sa) obtained from the three-dimensional surface profiles was used to describe the surface topographical variation. Among the investigated samples, the PEF-10 film exhibits the largest surface height variation, with an Sa value of 0.588 μm. This relatively high Sa value indicates the presence of more pronounced surface irregularities, which may be associated with heterogeneous phase distribution and surface structure evolution during the film formation process. After the incorporation of PE wax and SiO_2_, the size and number of surface protrusions are reduced, accompanied by a decrease in Sa values, suggesting a more homogeneous surface topographical feature of the composite films.

For PEH-1, although the large surface protrusions observed on the PEF-10 film are significantly reduced, the Sa value remains relatively high at 0.519 μm. This result suggests that the incorporation of a low amount of PE wax has a limited effect on modifying the surface topographical features of the composite film. When the PE wax content increases to 3 wt% in PEH-2, the surface becomes smoother and more homogeneous, and the Sa value decreases to 0.328 μm, representing a reduction of approximately 36.8% compared with PEH-1. The decreased Sa value indicates reduced surface height variation, which may be related to improved melt processability and more uniform film formation during processing. When the SiO_2_ content increases from PEH-1 to PEH-3, the Sa value slightly increases to 0.473 μm. This change may be associated with the increased contribution of inorganic nanoparticles to the surface topographical features and the formation of localized microscale structures [12]. Nevertheless, the Sa value remains lower than those of PEF-10 and PEH-1, suggesting that the presence of PE wax helps maintain relatively homogeneous surface characteristics. For PEH-4, the Sa value decreases again to 0.351 μm, indicating that the combined regulation of PE wax and SiO_2_ contents contributes to the formation of a more uniform surface topography.

The variation in surface topographical characteristics may further influence the water droplet spreading behavior of the composite films; therefore, the relationship between surface morphology and wettability is discussed in the following section.

The surface SEM-EDS elemental mapping images in Figure 2C provide information regarding the spatial distribution of F and Si elements on the observed film surfaces. For PEH-1, containing 1 wt% PE wax and 1 wt% SiO_2_, several F-rich regions can still be observed, although their size and intensity are reduced compared with those in the PEF-10 film. This observation suggests that the introduction of PE wax and SiO_2_ is associated with changes in the distribution of fluorine-containing regions, while localized heterogeneity remains. Meanwhile, several Si-rich regions are observed, indicating that the silica-containing domains are not completely homogeneous at this composition.

With increasing PE wax content from PEH-1 to PEH-2, the large F-rich regions become less pronounced, and the F signal exhibits a more homogeneous distribution in the observed areas. Meanwhile, the localized Si-rich regions are also reduced. These observations are consistent with the changes in surface topographical features observed by CLSM, suggesting that PE wax may promote a more uniform microstructural development during melt processing. This effect may be related to the improved melt processability of the composite system. When the SiO_2_ content increases from PEH-1 to PEH-3, no obvious enlargement of Si-rich regions is observed, and the distribution of F elements remains relatively homogeneous. This result suggests that the presence of PE wax helps reduce pronounced elemental heterogeneity during processing. The combined regulation of PE wax and SiO_2_ contents may contribute to the formation of more uniform observed surface regions.

Overall, the FTIR, CLSM, and EDS results confirm the successful incorporation of FEP, hydrophobic SiO_2_, and PE wax into the PE matrix through melt blending. Among the investigated formulations, increasing the PE wax content resulted in decreased surface height variation and more homogeneous elemental distribution in the observed regions, indicating improved surface structural uniformity of the composite films. These structural changes may contribute to the variations in water vapor barrier performance, gas transport behavior, optical properties, and mechanical properties discussed in the following sections.

### 3.2. Thermal Behavior and Surface Properties of PE-Based Composite Films

The macroscopic appearance and optical properties of the composite films are presented in Figure 3B,C. All films exhibit continuous and intact surfaces without visible cracks or macroscopic defects, indicating that the incorporation of PE wax and hydrophobic SiO_2_ does not significantly affect the film-forming capability of the PE/FEP system during melt processing. Compared with PEF-10, the PEH films exhibit a more uniform visual appearance, which may be associated with changes in surface morphology and phase distribution after multicomponent modification. The films show a milky or translucent appearance rather than high optical transparency, indicating that the incorporation of FEP, PE wax, and SiO_2_ influences the optical characteristics of the polyethylene matrix. The differences in optical transmittance among different formulations may be related to variations in phase morphology and refractive index contrast between the polymer matrix and inorganic components, which can affect light scattering behavior [24].

The melting and crystallization behaviors of PEF-10 and PE-based composite films were investigated by differential scanning calorimetry (DSC), as shown in Figure 4a,b. The corresponding thermal parameters and degree of crystallinity values are summarized in Table 2. All samples exhibit similar melting temperatures (Tm, approximately 120–122 °C) and crystallization temperatures (Tc, approximately 106–108 °C), indicating that the incorporation of PE wax and SiO_2_ does not significantly alter the fundamental melting and crystallization characteristics of the LLDPE phase. However, slight variations in melting enthalpy (ΔHm) and degree of crystallinity (Xc) are observed among different formulations. Compared with PEF-10, the PEH samples exhibit slightly lower degree of crystallinity values, decreasing from 20.54% for PEF-10 to 18.04–19.05% for PEH films. Considering the relatively small variation, the results suggest that the incorporation of FEP, PE wax, and SiO_2_ slightly modifies the crystallization behavior of the LLDPE matrix rather than causing a significant change in the crystalline structure.

The cooling curves provide further information regarding the crystallization behavior of the composite films. All samples exhibit similar crystallization temperatures (Tc, approximately 106–108 °C), indicating that the incorporation of FEP, PE wax, and SiO_2_ does not significantly affect the overall crystallization kinetics of the LLDPE phase. A weak shoulder observed in the cooling curves may be associated with the coexistence of different crystallization processes arising from the LLDPE matrix and the introduced functional components. However, no obvious shift in the main crystallization peak is observed, suggesting that the additives only moderately influence the crystallization process under the investigated conditions.

PE wax, as a low-molecular-weight polyolefin additive, may act as a processing aid and plasticizing component in the polyethylene matrix by increasing chain mobility and reducing melt resistance during processing. Meanwhile, owing to its polyethylene-like molecular structure, PE wax may also participate in crystallization behavior through the formation of its own crystalline regions. Therefore, the final degree of crystallinity of the composite films is determined by the balance between enhanced chain mobility, possible wax crystallization, and the restriction effects introduced by FEP and SiO_2_.

The PEH formulations exhibit only slight differences in degree of crystallinity. PEH-3 shows a relatively higher degree of crystallinity (19.05%), while PEH-4 exhibits the lowest value (18.04%). These variations are within a limited range, suggesting that the incorporation of PE wax and SiO_2_ slightly modifies the crystallization behavior of the PE matrix rather than inducing substantial changes in the crystalline structure. Overall, the DSC results demonstrate that the multicomponent modification strategy regulates the thermal behavior of the composite films while maintaining the intrinsic crystallization characteristics of the LLDPE phase.

The surface wettability of the composite films was evaluated using water and diiodomethane as probe liquids. The water contact angle (WCA) values of the films are presented in Figure 4c,d. The PEF-10 film exhibits a WCA of approximately 106.04°, while the optimized PEH film reaches 110.75°, indicating enhanced surface hydrophobicity after multicomponent modification. This improvement is mainly attributed to the contribution of low-surface-energy fluorinated groups from FEP and the hydrophobic characteristics of modified SiO_2_. Since PE wax possesses a chemical structure similar to polyethylene, it is not expected to directly increase the intrinsic hydrophobicity of the film surface. Instead, PE wax may influence film formation behavior and surface topographical characteristics, which can indirectly affect water droplet spreading.

According to the Cassie–Baxter model, surface topographical features can influence [20,21] the apparent contact angle by introducing trapped air at the solid–liquid interface. However, the Sa values obtained from CLSM do not show a direct monotonic relationship with WCA among the PEH films. For example, PEH-2 exhibits the highest WCA despite not having the largest Sa value. This indicates that surface chemical composition, particularly the presence of fluorinated groups and hydrophobic SiO_2_, plays a dominant role in determining the hydrophobic behavior, while surface topographical variation provides an additional contribution.

Overall, the thermal and wettability results indicate that the incorporation of PE wax and SiO_2_ influences both the crystallization behavior of the PE matrix and the surface properties of the composite films. The adjustment of functional component contents enables the regulation of surface hydrophobicity and crystallization behavior, providing a basis for further optimization of gas transport and water vapor barrier performance.

### 3.3. Water Vapor Barrier and CO_2_ Transport Properties of PE-Based Composite Films

(1) Water vapor resistance and CO_2_ transport properties

The water vapor resistance and CO_2_ transport properties of the PE-based composite films are summarized in Table 3 and Figure 5a. The fabricated films are dense non-porous polyethylene-based composite membranes prepared by melt blending and film casting. Therefore, gas transport is expected to occur mainly through a solution–diffusion mechanism rather than through interconnected pores [1,4]. In this mechanism, gas transport is governed by the dissolution of penetrant molecules into the polymer matrix and their subsequent diffusion through polymer free volume. The permeability coefficient (P) can be described as:

P = D × S(2)

where D represents the diffusion coefficient and S represents the solubility coefficient. In contrast, the transmission rate represents the amount of gas or vapor passing through a membrane with a given thickness under specific testing conditions. Therefore, transmission rate values reported in this work are used to compare the practical transport performance of the composite films.

The PEF-10 film exhibits a water vapor transmission rate (WVTR) of 1.80 g m^−2^ day^−1^ and a water vapor permeability (WVP) of 8.317 × 10^−15^ g·cm cm^−2^ s^−1^ Pa^−1^. After incorporating PE wax and hydrophobic SiO_2_, the moisture barrier performance of the composite films is improved.

As the PE wax content increases from PEH-1 to PEH-2, the WVTR decreases from 1.60 to 1.20 g m^−2^ day^−1^, corresponding to a reduction of approximately 25%. In comparison, increasing the SiO_2_ content from PEH-1 to PEH-3 decreases the WVTR from 1.60 to 1.40 g m^−2^ day^−1^, representing a reduction of approximately 12.5%. These results indicate that, under the investigated compositions, PE wax has a stronger influence on reducing water vapor transmission, while SiO_2_ provides an additional contribution to moisture barrier performance.

The improved water vapor resistance can be attributed to the combined effects of reduced water affinity and increased diffusion resistance [5,11,12]. The fluorinated segments in FEP provide low-polarity fluorocarbon groups, reducing the interaction between water molecules and the membrane surface. Hydrophobic SiO_2_ further enhances surface water repellency and may increase the tortuosity of diffusion pathways. PE wax, due to its low-molecular-weight polyolefin structure, may modify chain mobility and film formation behavior, thereby influencing the diffusion process of water vapor through the dense polymer matrix.

Although the composite films are dense and hydrophobic, the WVTR values are not zero because water vapor molecules can still dissolve into the polymer phase and diffuse through transient free-volume pathways according to the solution–diffusion mechanism. Therefore, the incorporation of FEP, PE wax, and SiO_2_ provides an effective approach for regulating moisture barrier performance of polyethylene-based composite films.

The CO_2_ transport properties of the composite films are summarized in Table 3 and Figure 5a. The PEF-10 film exhibits a CO_2_ transmission rate of 2895 cm^3^ m^−2^ day^−1^ at 0.1 MPa. After incorporating PE wax and hydrophobic SiO_2_, the CO_2_ transmission rate increases significantly, reaching 8245 cm^3^ m^−2^ day^−1^ for PEH-1, corresponding to an increase of approximately 184.8%. The increased CO_2_ transmission rate after multicomponent modification can be explained by changes in both diffusion and solubility contributions. The introduction of FEP, PE wax, and SiO_2_ creates heterogeneous polymer-additive interfaces, which may alter local chain packing and provide additional pathways for CO_2_ diffusion. Meanwhile, CO_2_ has a higher condensability and quadrupole interaction compared with nonpolar gases, allowing stronger interactions with polarizable polymer segments and fluorinated regions. Therefore, changes in polymer-gas affinity may also contribute to the variation in CO_2_ transport behavior.

However, further increasing the PE wax content (PEH-1 to PEH-2) decreased the CO_2_ permeability by approximately 21.2%, which may be attributed to partial occupation of diffusion pathways by excessive wax domains. Similarly, increasing the SiO_2_ content (PEH-1 to PEH-3) reduced CO_2_ permeability by approximately 21.9%, likely due to increased diffusion resistance caused by higher inorganic loading. When both PE wax and SiO_2_ contents were increased simultaneously (PEH-4), the CO_2_ permeability further decreased to 5004 cm^3^ m^−2^ day^−1^ 0.1 MPa^−1^.

To quantitatively evaluate the balance between CO_2_ transport capability and water vapor resistance, the CO_2_ permeability/WVTR ratio was calculated according to Equation (2) ($P_{{CO}_{2}}$: CO_2_ permeability; WVTR: water vapor transmission rate). The PEH films exhibited significantly higher ratios than PEF-10, with the maximum value reaching 5415 for PEH-2. The CO_2_ transmission rate/WVTR ratio was used as a comparative parameter to evaluate the balance between CO_2_ transport capability and moisture resistance. The higher ratio observed for PEH films indicates that CO_2_ transport is maintained while water vapor transmission is effectively reduced. This behavior originates from the different interactions of CO_2_ and water molecules with the hydrophobic polymer matrix. Water vapor transport is restricted by the low water affinity of fluorinated and hydrophobic components, whereas CO_2_ transport is less affected due to its higher affinity for the polymer phase and greater diffusivity.

(3) $$R_{{CO}_{2}/WVTR}=\frac{T_{{CO}_{2}}}{WVTR}$$

Compared with previously reported polyethylene-based barrier films, the present composite membranes exhibit a relatively low WVTR while maintaining a high CO_2_ transmission capability, demonstrating an improved balance between moisture resistance and gas transport performance [2,6]. This balanced transport behavior is attributed to the synergistic effects of hydrophobic fluorinated components, silica-induced interfacial regulation, and maintained CO_2_ transport capability of the polyethylene matrix. Unlike porous membranes, where transport behavior is strongly influenced by pore size and connectivity, the present dense non-porous membranes mainly rely on differences in molecular solubility and diffusion behavior within the polymer matrix.

(2) Optical properties

The optical properties of the PE-based composite films are presented in Figure 3 and Figure 5b. Compared with PEF-10, the PEH films exhibit increased optical transmittance while maintaining relatively high haze values. The light transmittance increases from 76.48% for PEF-10 to 84.60–85.79% for PEH films, indicating that multicomponent modification influences the light transmission behavior of the composite films. This improvement may be associated with changes in phase morphology and surface structural characteristics after PE wax incorporation, which can reduce excessive light scattering within the composite films [12].

Meanwhile, the haze values remain relatively high but show an overall decreasing trend, with PEH-2 exhibiting the lowest haze value of 93.60%. The relatively high haze values indicate that the films still exhibit significant light scattering characteristics, consistent with their milky appearance. The variation in haze among different formulations may be related to differences in phase morphology, refractive index contrast between polymer components and silica nanoparticles, and surface structural changes. Therefore, the incorporation of PE wax and SiO_2_ modifies the optical properties of the PE/FEP composite films without resulting in highly transparent films.

(3) Mechanical properties

The mechanical properties of the PE-based composite films are presented in Figure 5c,d. The tensile stress–strain curves provide information regarding the elastic deformation, yielding behavior, plastic deformation, and final fracture process of the composite films. Compared with PEF-10, the incorporation of PE wax and SiO_2_ influences both the strength and ductility of the PE matrix. The tensile strength increases from 9.75 MPa for PEF-10 to 10.55 MPa for PEH-1, while the elongation at break increases from 690% to 835%. The changes observed in the yielding and post-yield deformation regions indicate that the incorporated functional components modify the deformation behavior of the polyethylene matrix.

Further increasing the PE wax content from PEH-1 to PEH-2 increases the tensile strength to 12.21 MPa and the elongation at break to 914%. This improvement may be related to the plasticizing effect of PE wax, which increases polymer chain mobility and facilitates plastic deformation during tensile loading. The reduced resistance to chain rearrangement contributes to enhanced ductility, while the polyethylene-like structure of PE wax maintains compatibility with the matrix and avoids severe loss of mechanical integrity.

Increasing the SiO_2_ content from PEH-1 to PEH-3 also improves the mechanical performance. The silica nanoparticles may provide reinforcement through interactions with the polymer matrix and restrict excessive chain movement, thereby improving resistance to deformation. However, excessive inorganic loading may increase interfacial constraints and reduce molecular mobility.

Overall, the incorporation of PE wax and hydrophobic SiO_2_ improves the mechanical performance of the PE/FEP composite films. PE wax mainly regulates chain mobility and deformation behavior, whereas SiO_2_ contributes to reinforcement through polymer–filler interactions. The combined modification strategy enables a balance between strength and flexibility, which is important for the practical application of polyethylene-based functional films.

## 4. Conclusions

In summary, polyethylene-based composite films were successfully fabricated through a scalable melt blending and film casting process by incorporating fluorinated ethylene propylene copolymer (FEP), polyethylene wax (PE wax), and hydrophobic SiO_2_ nanoparticles. The synergistic effects of these functional components effectively regulated the surface properties, interfacial morphology, crystallization behavior, and transport characteristics of the composite films, resulting in an improved balance between water vapor barrier performance and CO_2_ transport capability.

Among the investigated formulations, PEH-2 exhibited the most balanced overall performance, with a water vapor transmission rate (WVTR) of 1.20 g m^−2^ day^−1^, a CO_2_ transmission rate of 6498 cm^3^ m^−2^ day^−1^, and a CO_2_ transmission rate/WVTR ratio of approximately 5.4 × 10^3^. The enhanced performance is attributed to the synergistic regulation of surface energy, additive dispersion, and interfacial structure by FEP, PE wax, and hydrophobic SiO_2_. These findings provide an effective and scalable strategy for designing polyethylene-based composite membranes with balanced moisture barrier and gas transport properties.

## Figures and Tables

**Figure 1:**
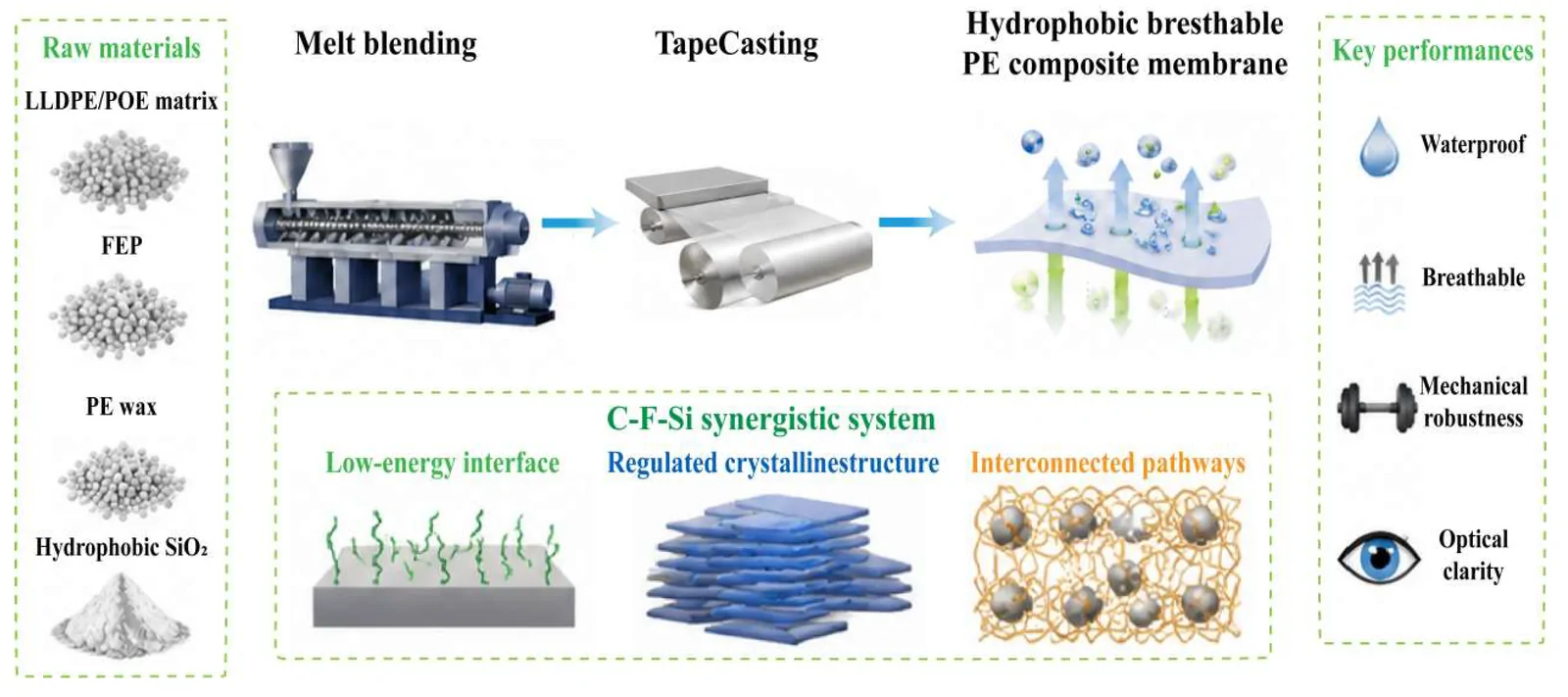
Schematic illustration of the proposed multicomponent modification strategy and the expected effects of FEP, PE wax, and SiO_2_ on surface properties, interfacial morphology, and transport behavior of the polyethylene matrix.

**Figure 2:**
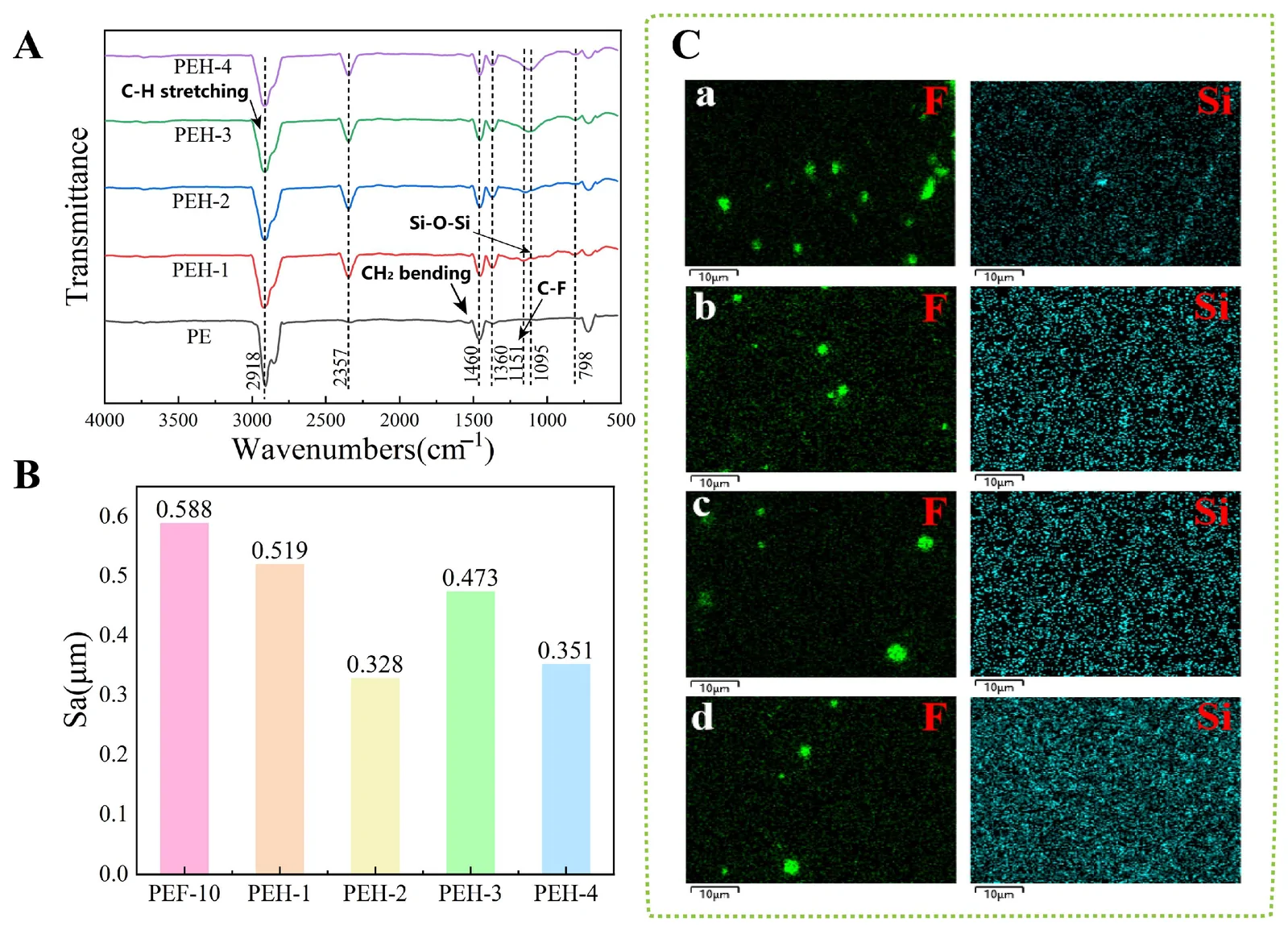
Structural characterization and surface morphology of PE composite films. (**A**) FTIR spectra of PE and PEH membranes. (**B**) Surface topographical variation (Sa) values of different composite films. (**C**) Surface SEM-EDS elemental mapping images showing the spatial distribution of F and Si elements in the observed regions (**a**) PEH-1 (**b**) PEH-2 (**c**) PEH-3 (**d**) PEH-4.

**Figure 3:**
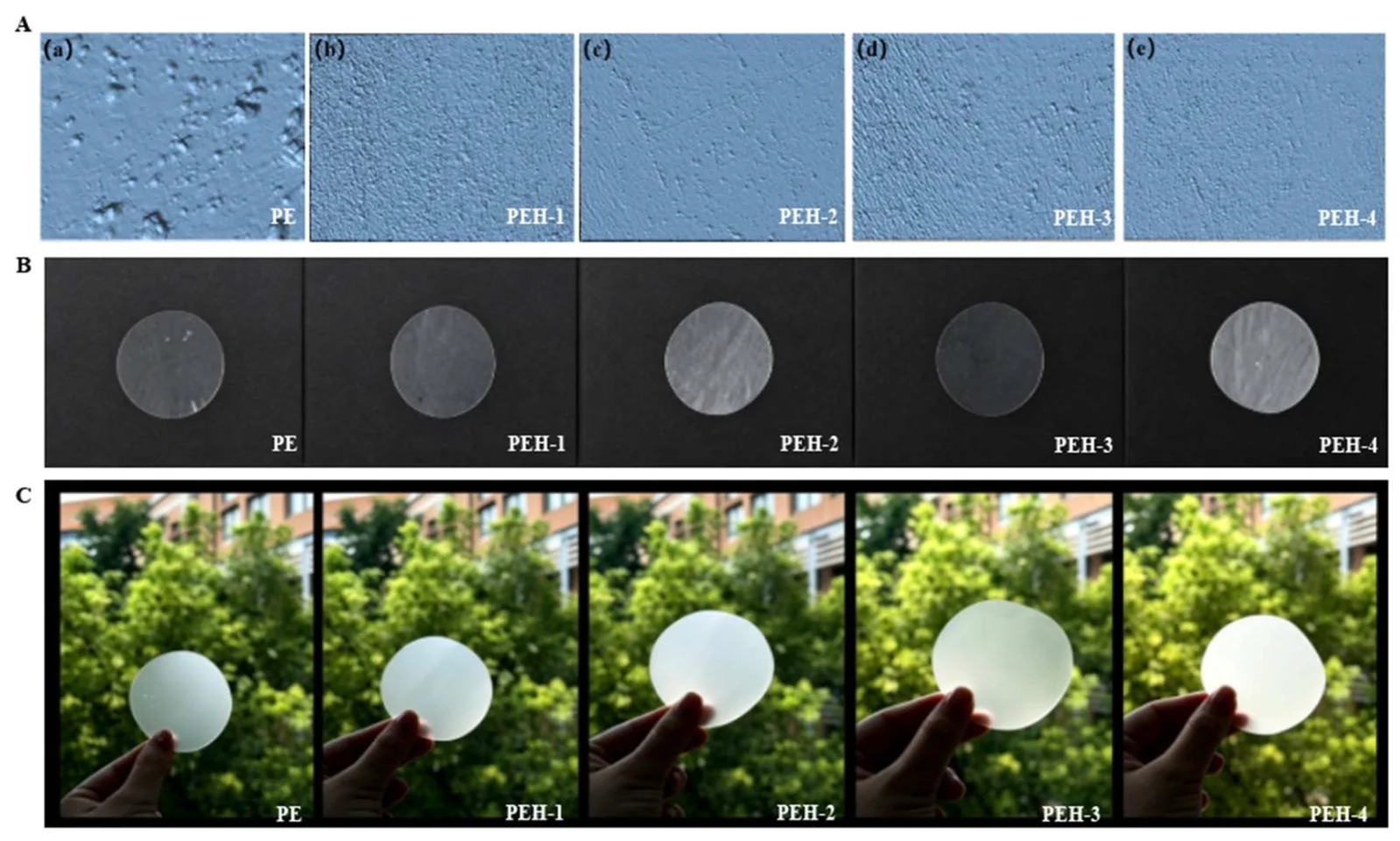
Surface topographical features and optical properties of PEH films. (**A**) CLSM images showing the three-dimensional surface morphology of PE and PEH films with different compositions. (**B**) Digital photographs of the corresponding films. (**C**) Optical transmittance spectra of the films against a natural background: (**a**) PE; (**b**) PEH-1; (**c**) PEH-2; (**d**) PEH-3; (**e**) PEH-4.

**Figure 4:**
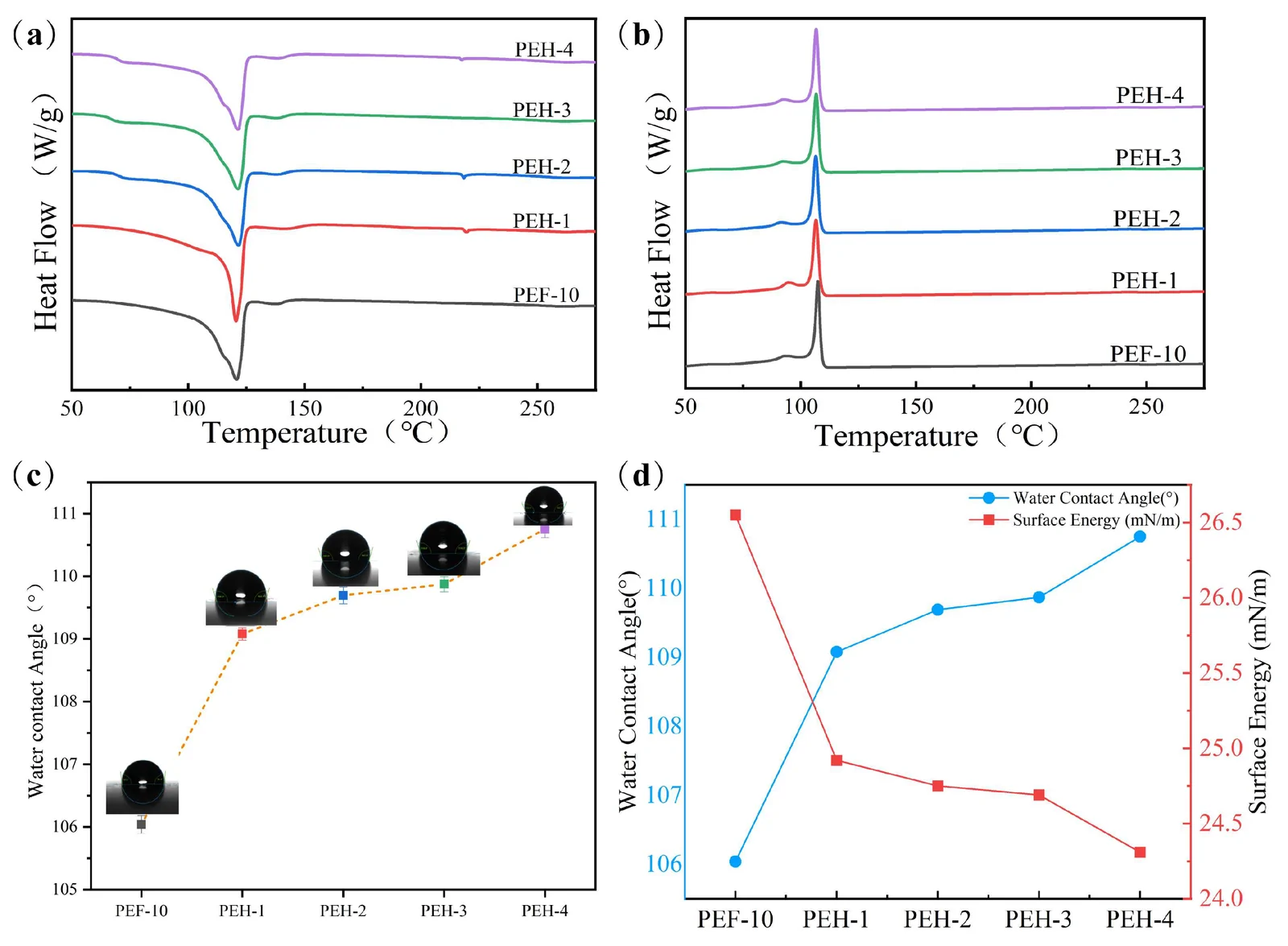
Thermal behavior and surface wettability of PEF-10 and PEH films. (**a**) DSC melting curves during heating. (**b**) DSC crystallization curves during cooling. (**c**) Water contact angle images and corresponding surface energy values of the composite films, (**d**) Changes in water contact angle and surface energy of PEF-10 and PEH films with different compositions.

**Figure 5:**
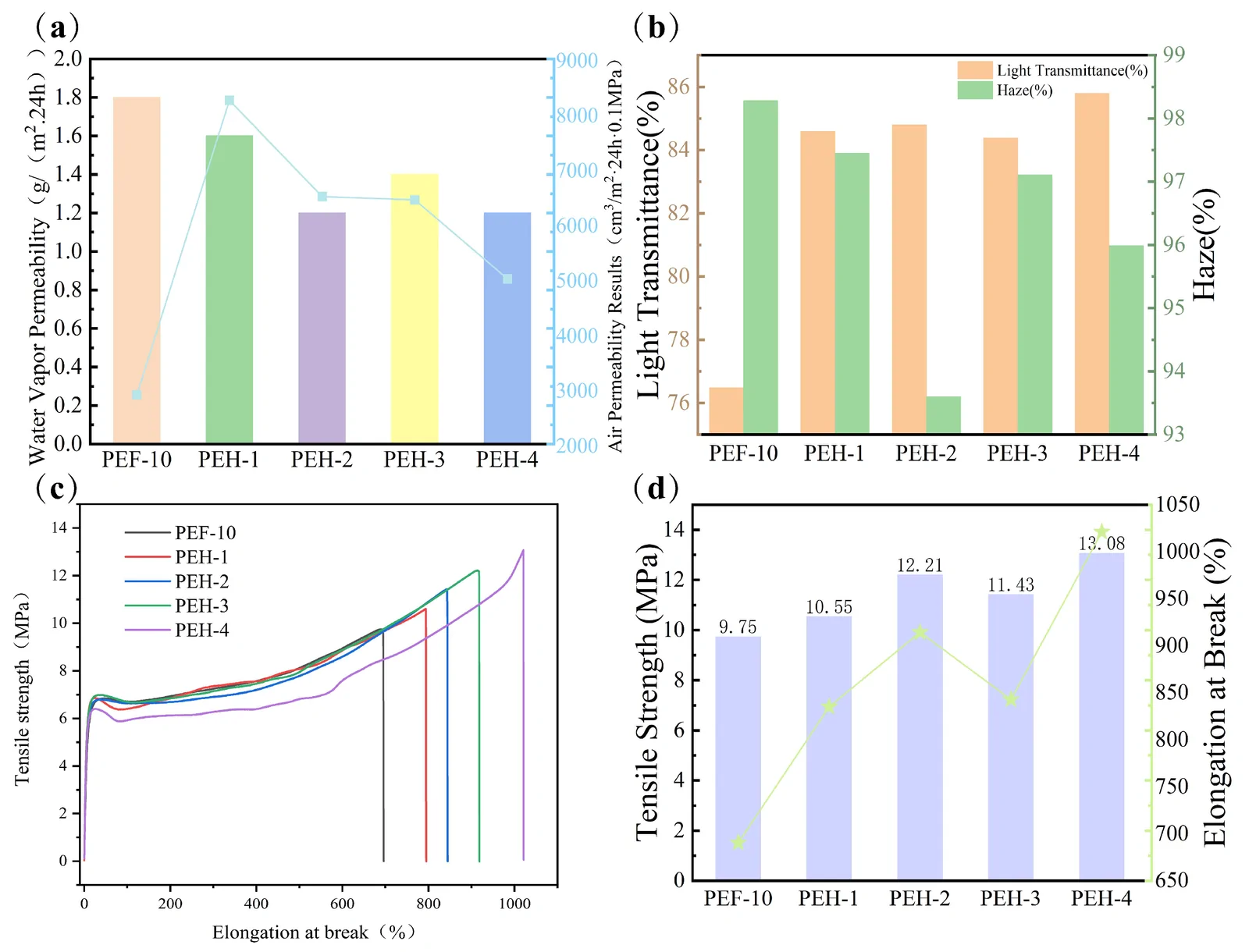
Barrier, optical, and mechanical properties of PEF-10 and PEH films. (**a**) Water vapor transmission rate and CO_2_ transmission rate. (**b**) Light transmittance and haze. (**c**) Representative tensile stress–strain curves. (**d**) Tensile strength and elongation at break of PEF-10 and PEH films.

**Table 1:** Composition of PE/FEP/PE wax/SiO_2_ composite films.

Sample Name	LLDPE	POE	FEP	PEWax	SiO_2_
PEF-10	70	30	10	--	--
PEH-1	70	30	10	1	1
PEH-2	70	30	10	3	1
PEH-3	70	30	10	1	3
PEH-4	70	30	10	3	3

The contents of PE wax and SiO_2_ are calculated as weight percentages (wt%) relative to the total mass of the LLDPE/POE matrix.

**Table 2:** Thermal parameters and degree of crystallinity of PEF-10 and PEH composite films.

Sample	Tm (°C)	Tc (°C)	ΔHm (J g^−1^)	Xc (%)
PEF-10	121.16 ± 0.35	107.59 ± 0.62	42.12 ± 0.50	20.54 ± 0.24
PEH-1	120.46 ± 0.35	106.60 ± 0.35	38.46 ± 0.50	18.75 ± 0.24
PEH-2	121.43 ± 0.35	106.52 ± 0.35	38.53 ± 0.50	18.79 ± 0.24
PEH-3	121.31 ± 0.35	106.85 ± 0.35	39.08 ± 0.50	19.05 ± 0.24
PEH-4	121.24 ± 0.35	106.64 ± 0.35	37.01 ± 0.50	18.04 ± 0.24

**Table 3:** Water vapor barrier and gas transport properties of PEF-10 and PEH films.

Sample	WVTR	CO_2_ Transmission Rate (cm^3^ m^−2^ day^−1^)	CO_2_ Transmission Rate/WVTR Ratio
PEF-10	1.80	2895 ± 50	1608
PEH-1	1.60	8245 ± 50	5158
PEH-2	1.20	6498 ± 50	5415
PEH-3	1.40	6436 ± 50	4597
PEH-4	1.20	5004 ± 50	4170

The CO_2_ transmission rate/WVTR ratio was calculated from the measured CO_2_ transmission rate and WVTR values.

**Figure A1:**
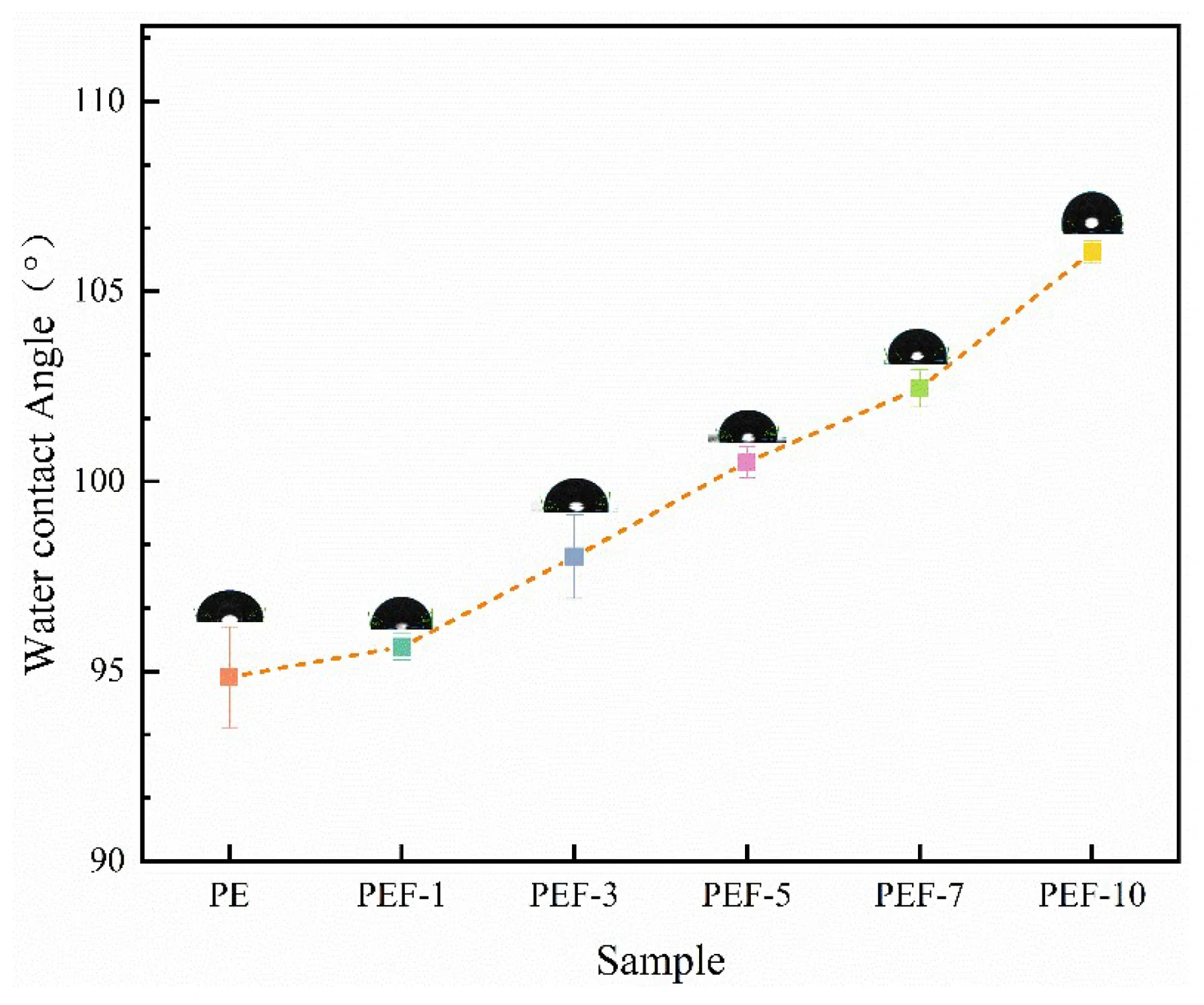
Water contact Angle curve of the PEF film.

**Figure A2:**
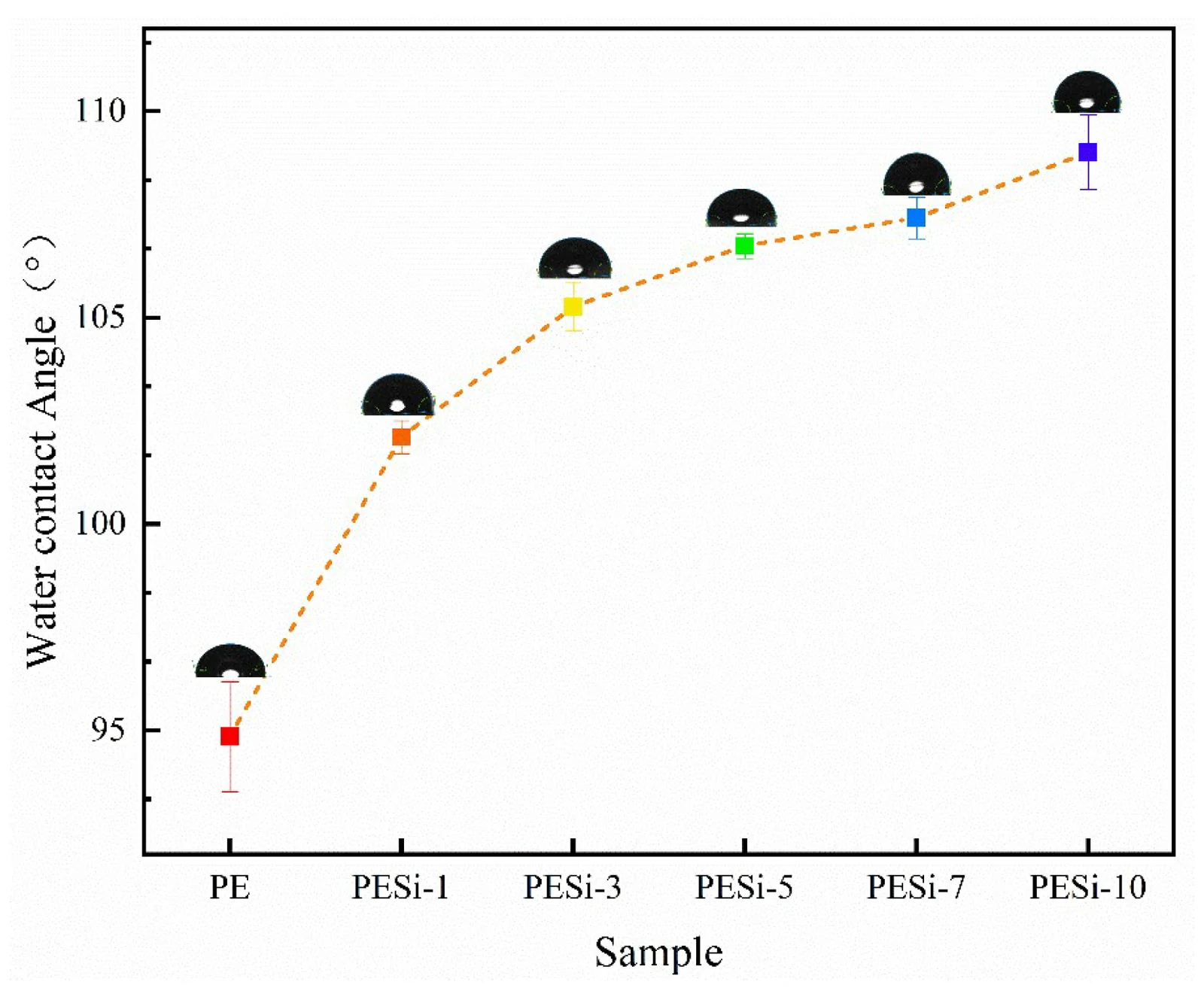
Water contact Angle curve of the PESi film.

**Figure A3:**
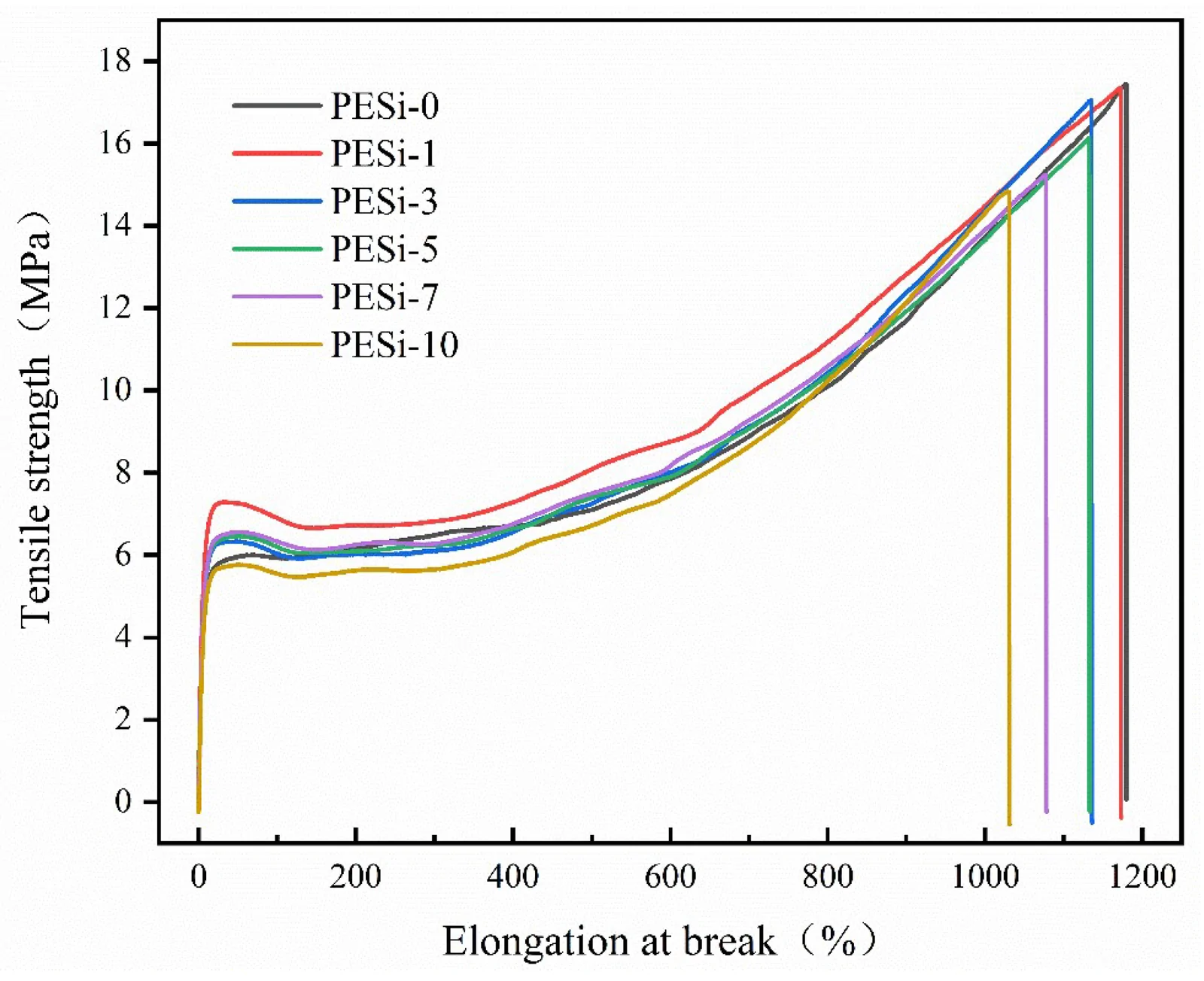
Stress–strain curves of PESi films.

## Data Availability

The data presented in this study are available upon request from the corresponding author.

## Appendix A

Preparation of FEP Composite Materials

The FEP-based composite materials were prepared via a melt blending process. Linear low-density polyethylene (LLDPE), polyolefin elastomer (POE), and fluorinated ethylene propylene copolymer (FEP) were first weighed according to the compositions listed in Table A1 and subjected to dry mixing in a high-speed mixer for 3–5 min to ensure homogeneous dispersion of the components. The premixed materials were subsequently fed into a co-rotating twin-screw extruder for melt compounding. The temperature profile of the extruder was set as follows: 265 °C for the first zone, 280 °C for the second zone, 275 °C for the third zone, and 270 °C for the die. The molten blend was extruded through the die, rapidly cooled in a water bath, dried by air circulation after traction, and pelletized using a rotary pelletizer to obtain the FEP composite pellets.

Fabrication of PEF Composite Films

The as-prepared composite pellets were dried in a forced-air oven at 80 °C for 1 h to remove residual moisture. The dried pellets were then processed into films through melt extrusion followed by a casting process. Specifically, the composite pellets were fed into a single-screw extruder equipped with a flat film die, where they were melted and homogenized under controlled processing conditions. The molten polymer melt was subsequently extruded through the flat die and cast onto a cooled casting roll to form continuous composite films. The thickness of the films was controlled by adjusting the extrusion rate and casting speed. After cooling and solidification, the obtained films were collected and further conditioned at room temperature prior to characterization. This melt extrusion-casting process enabled the preparation of uniform PEF composite films with well-dispersed FEP domains and reproducible thickness.

**Table A1:** Blending ratio of LLDPE, POE and FEP.

Sample Name	LLDPE/(wt%)	POE/(wt%)	FEP/(wt%)
PE	70	30	--
PEF-1	70	30	1
PEF-3	70	30	3
PEF-5	70	30	5
PEF-7	70	30	7
PEF-10	70	30	10

Contact Angle of PEF Composite Films

WVTR of PEF Composite Films

**Table A2:** Water vapor transmission data of PEF films.

Name	PE	PEF-1	PEF-3	PEF-5	PEF-7	PEF-10
Film Thickness (μm)	167	147	163	198	137	184
Water Vapor Permeability (g/(m^2^·24 h))	10.74	3.60	2.80	2.40	2.20	1.80
Water Vapor Permeability Coefficient (g.cm/cm^2^·s·Pa)	3.188 × 10^−14^	9.191 × 10^−15^	8.562 × 10^−15^	8.600 × 10^−15^	8.575 × 10^−15^	8.317 × 10^−15^
Breathability Results (cm^3^/m^2^·24 h·0.1 MPa)	3763	6315	6129	5118	4014	2895
Air-to-Water Permeability Ratio	350	1754	2188	2132	1824	1608

Optical transmission of PEF Composite Films

**Table A3:** Optical transmission data of the PEF film.

Name	PE	PEF-1	PEF-3	PEF-5	PEF-7	PEF-10
Light Transmittance (%)	84.86	82.55	81.26	80.30	78.60	76.48
Haze (%)	42.47	97.91	98.06	98.19	98.20	98.28
Film Thickness (μm)	167	147	163	198	137	184

Preparation of FESi Composite Materials

In this experiment, the melt blending method was used to prepare polymer composites. The specific process flow is as follows: First, linear low-density polyethylene (LLDPE), polyolefin elastomer (POE), and silicon dioxide (SiO_2_) were placed in a high-speed mixer in the mass ratios shown in Table A4 for dry mixing. The mixing time was controlled at 3–5 min to ensure uniform dispersion of the materials. The premix was then fed into a co-rotating twin-screw extruder for melt extrusion blending. The processing temperature parameters were set as follows: Zone 1 at 160 °C, Zone 2 at 180 °C, and Zone 3 at 170 °C, with the die temperature maintained at 165 °C. After being extruded through the die, the melt is rapidly cooled and shaped via a water-cooling system. Following air-drying in the haul-off unit, it enters a rotary pelletizer for pelletization, ultimately yielding the blended pellets.

Fabrication of PESi Composite Films

The blended particles were dried in a forced-air drying oven at a constant temperature of 80 °C for 1 h to remove residual moisture, and then transferred to the mold cavity of a flat-plate vulcanizer. The temperatures of the upper and lower heating plates were both set to 175 °C, and the material was hot-pressed at a pressure of 30 MPa. After holding the pressure, the material was shaped using a gradient cooling system, ultimately producing the composite film.

**Table A4:** Blending ratio of LLDPE, POE and SiO_2._

Sample Name	LLDPE (wt%)	POE (wt%)	SiO_2_ (wt%)
PE	70	30	--
PESi-1	70	30	1
PESi-3	70	30	3
PESi-5	70	30	5
PESi-7	70	30	7
PESi-10	70	30	10

**Table A5:** Roughness data of films with different SiO_2_ additions.

Sample Name	PE	PESi-1	PESi-3	PESi-5	PESi-7	PESi-10
Sa (μm)	0.216	0.235	0.280	0.337	0.419	0.498

Contact Angle of PESi Composite Films

WVTR of PESi Composite Films

**Table A6:** Water vapor transmission data of PESi films.

Sample Name	PE	PESi-1	PESi-3	PESi-5	PESi-7	PESi-10
Film Thickness (μm)	167	147	200	181	164	200
Water Vapor Permeability (g/(m^2^·24 h))	10.74	3.20	2.80	2.80	2.40	2.00
Water Vapor Permeability Coefficient (g.cm/cm^2^·s·Pa)	3.188 × 10^−14^	9.191 × 10^−15^	9.946 × 10^−15^	9.946 × 10^−15^	8.137 × 10^−15^	4.361 × 10^−15^
Breathability Results (cm^3^/m^2^·24 h·0.1 MPa)	3763	5928	5523	4972	4323	4046
Air-to-Water Permeability Ratio	350	1852	1972	1775	1801	2023

Mechanical Properties of PESi Composite Films

Optical transmission of PESi Composite Films

**Table A7:** Optical transmission data of the PESi film.

Name	PE	PESi-1	PESi-3	PESi-5	PESi-7	PESi-10
Light Transmittance (%)	84.86	85.11	85.57	86.13	86.43	86.98
Haze (%)	42.47	44.31	44.67	44.94	45.11	45.63
Film Thickness (μm)	167	147	200	181	164	200

## References

[1] Chang Y., Liu Y., Liu F. (2024). Simple fabrication of breathable poly (vinylidene fluoride) fibrous membranes decorated by silica nanoparticles with enhanced waterproof property. Ceram. Int..

[2] Wang H., Li J., Chen G., Zhong Y., Cheng Z., Zhang C., Zhao P., Yang J., Xiao N. (2024). Hydrophobic polyethylene film prepared by film blowing process for preservation of fried shrimp rolls. Food Chem..

[3] Liu Q., Li Z., Lu T., Zhu Q., Mao X., Zhu L., Wang X., Zhu M. (2025). High-density polyethylene Janus fibrous membrane with enhanced breathability and moisture permeability via PDA-assisted hydrophilic modification. Macromol. Rapid Commun..

[4] Zhao J., Zhang T., Li Y., Huang L., Tang Y. (2023). Fluorine-free, highly durable waterproof and breathable fibrous membrane with self-clean performance. Nanomaterials.

[5] Lee J., Yeasmin S., Jung J.H., Kim T.Y., Kwon T.Y., Kwon D.Y., Yeum J.H. (2023). Preparation and characterization of thermal-insulating microporous breathable Al/LLDPE/CaCO3 composite films. Materials.

[6] Luo T., Farooq A., Weng W., Lu S., Luo G., Zhang H., Li J., Zhou X., Wu X., Huang L. (2024). Progress in the preparation and application of breathable membranes. Polymers.

[7] Zhang H., Zhai Q., Cao Y., Hu J., Zhen Q., Qian X. (2023). Design and facile manufacturing of tri-layer laminated polyolefin microfibrous fabrics with tailoring pore size for enhancing waterproof breathable performance. Mater. Des..

[8] Liu S., Jun S.C., Zhang S., Wang F., Yu J., Ding B. (2024). Advancements in electrospun nanofibrous membranes for improved waterproofing and gas permeability. Macromol. Mater. Eng..

[9] Hu S., Ren G., Zhang Y., Liu Y., Zhang W., Sun W., Li D., Cui Z., Yu W., Lu D. (2025). Fluorine-free, superhydrophobic polyurethane and stearic acid waterproof and breathable electrospun nanofiber membrane with excellent hydrostatic pressure resistance. Langmuir.

[10] Li Y., Gu J., Cao K., Qin X. (2024). Effects of fluorosilane addition on polyurethane electrospun membranes with waterproof and breathable performance. Text. Res. J..

[11] Babin M., Eder G.C., Voronko Y., Oreski G. (2024). Water vapor permeability of polymeric packaging materials for novel glass-free photovoltaic applications. J. Appl. Polym. Sci..

[12] Zhao K., Zhang H., Zhai Q., Guan X., Zhen Q., Cui J. (2024). Scalable, waterproof, breathable, and flexible polyolefin-elastomer/polyethylene glycol@zinc oxide microfibrous fabrics for daytime radiative cooling clothing. ACS Appl. Mater. Interfaces.

[13] Song S.H., Bae M., Oh J.K. (2024). Durable Surface Modification of Low-Density Polyethylene/Nano-Silica Composite Films with Bacterial Antifouling and Liquid-Repelling Properties for Food Hygiene and Safety. Polymers.

[14] Li Z., Ren G., Feng Y., Zhang Y., Cui Z., Lu D., Zhang L., Yu H., Li W., Liu P. (2024). Electrospinning of fluorine-free waterproof–breathable nanofiber membranes with thermal regulation for anticounterfeiting. ACS Appl. Nano Mater..

[15] Hao W., Zhong Y., Yang Q., Ke C., Lu Y., Wang W., Yang W. (2024). Superhydrophobic and breathable polydimethylsiloxane/nano-SiO2@polylactic acid electrospun membrane with core-sheath fiber structure. Prog. Org. Coat..

[16] Xing X., Zhang X., Tasin M.A.S., Liang X., Zhou H., Niu H. (2024). Interlaced amphiphobic nanofibers for smart waterproof and breathable membranes with instant waterproofness monitoring ability. ACS Appl. Polym. Mater..

[17] Mao Q., Ding M., Yin X., Yu J., Zhang S., Ding B. (2024). Superelastic and fluorine-free nanofiber membranes prepared by one-step electrospinning for waterproof and breathable applications. Compos. Commun..

[18] Chen J., Yao N., Wang T., Wu J., Ding M., Zhang X., Zhang S., Yoon S.S., Ding B. (2024). High-performance fluorine-free waterproof and breathable electrospun nanofibrous membranes by thermally induced coating-crosslinking. Compos. Commun..

[19] Zhang Y., Ren G., Nie G., Hu S., Sun W., Li D., Cui Z., Li Y., Lu D., Yu H. (2025). One-step electrospinning for fluorine-free nanofiber membranes with optimally integrated waterproofing, gas permeability, and UV protection. ACS Appl. Polym. Mater..

[20] Li F., Weng K., Tanaka T., He J., Zheng H., Noda D., Irifune S., Sato H. (2024). Fabrication of waterborne silicone-modified polyurethane nanofibers for nonfluorine elastic waterproof and breathable membranes. Polymers.

[21] Lu D., Li Y., Chai Y., Wen X., Chen L., Sun S. (2025). Single-component silicon-containing polyurethane for high-performance waterproof and breathable nanofiber membranes. Fibers.

[22] Hojatzadeh S., Rahimpour F., Sharifzadeh E. (2024). Isothermal crystallization kinetics of pure and surface-modified silica/high-density polyethylene nanocomposites. Polym. Compos..

[23] Park Y., Kim J., Kim D., Lee S., Hwang D., Kwon M.S. (2024). Towards the optimal design of optically clear adhesives for flexible display. Soft Sci..

[24] Ma L., Amador E., Belev G.S., Gautam C., Zhou W., Liu J.P., Sammynaiken R., Chen W. (2024). Tuning Ag^+^ and Mn^2+^ doping in ZnS:Ag, Mn embedded polymers for flexible white light emitting films. Soft Sci..

